# Longitudinal determinants of 12-month changes on bone health in adolescent male athletes

**DOI:** 10.1007/s11657-018-0519-4

**Published:** 2018-10-10

**Authors:** Esther Ubago-Guisado, Dimitris Vlachopoulos, Ioannis G. Fatouros, Chariklia K. Deli, Diamanda Leontsini, Luis A. Moreno, Daniel Courteix, Luis Gracia-Marco

**Affiliations:** 1 IGOID Research Group, University of Castilla-La Mancha, Avenida Carlos III s/n, 45071 Toledo, Spain; 2 Children’s Health and Exercise Research Centre, Sport and Health Sciences, University of Exeter, 79 Heavitree Rd, Exeter, EX2 4TH United Kingdom; 3 School of Physical Education and Sport Sciences, Democritus University of Thrace, 69100 Komotini, Greece; 4 School of Physical Education and Sport Sciences, University of Thessaly, Karies, 421 00 Trikala, Greece; 5 GENUD Research Group, University of Zaragoza, C/Pedro Cerbuna 13, 50013 Zaragoza, Spain; 6 Laboratory of Metabolic Adaptations to Exercise in Physiological and Pathological conditions (AME2P), Université Clermont Auvergne, F-63000 Clermont-Ferrand, France; 7 Faculty of Health, School of Exercise Science, Australian Catholic University, 115 Victoria Parade Fitzroy, Victoria 3065 Australia; 8 PROFITH “PROmoting FITness and Health Through Physical Activity” Research Group, Department of Physical Education and Sport, Faculty of Sport Sciences, University of Granada, Carretera de Alfacar s/n, 18011 Granada, Spain

**Keywords:** Body composition, Bone mineral density, Hip geometry, Children, Exercise, Trabecular bone score

## Abstract

***Summary*:**

We identified the determinants of 12-month changes of areal bone mineral density (aBMD), hip geometry and trabecular bone score (TBS) in adolescent male athletes. Changes in region-specific lean mass and the type of sport are the most consistent determinants in this population.

**Purpose:**

This study aims to identify the determinants of 12-month changes of areal bone mineral density (aBMD), hip geometry and trabecular bone score (TBS) in adolescent male athletes.

**Methods:**

The sample was 104 adolescent males aged 12–14 years at baseline that were followed over 12 months: 39 swimmers, 37 footballers (or soccer players) and 28 cyclists. Dual-energy X-ray absorptiometry measured aBMD at the whole body, lumbar spine and dual hip. Hip geometry estimates at the femoral neck were measured using hip structural analysis. Lumbar spine texture was measured by TBS.

**Results:**

Multivariate regression models significantly explained 38–60% of the variance in the aBMD changes, 36–62% in the hip geometry estimates changes and 45% in the TBS changes. Δregion-specific lean mass was the most consistent predictor of changes in aBMD outcomes (*β* = 0.591 to 0.696), followed by cycling participation (*β* = − 0.233 to − 0.262), swimming participation (*β* = − 0.315 to − 0.336) and ΔMVPA (*β* = 0.165). Cycling participation was the most consistent predictor of changes in hip geometry estimates (*β* = − 0.174 to − 0.268), followed by Δregion-specific lean mass (*β* = 0.587) and Δcardiorespiratory fitness (*β* = 0.253). Finally, cycling and swimming participation (*β* = − 0.347 to − 0.453), Δregion-specific lean mass (*β* = 0.848) and Δstature (*β* = 0.720) were predictors of change in TBS.

**Conclusions:**

Changes in region-specific lean mass and the type of sport are the most consistent determinants of 12-month changes in aBMD, hip geometry estimates and TBS in adolescent male athletes.

**Trial registration:**

ISRCTN17982776

## Introduction

Among the different osteoporotic fractures, hip fracture is the one with the highest prevalence of mortality in the elderly population, due to a severe decline in bone mass with ageing [1]. Therefore, there is a need for early and effective preventive strategies. The greatest growth and skeletal maturation occurs at the end of puberty when ~ 51% of the peak bone mass is attained [2]. Genetic factors mainly contribute to the accumulation of bone mass accounting for 60 to 80% of the peak bone mass variance [3]. In addition, lifestyle factors such as physical activity [4] and the intake of calcium or vitamin D [5] can contribute to optimise peak bone mass [6]. Biological factors associated with bone growth vary significantly depending on level of maturity during adolescence, such as biological age [7].

Exercise during childhood has been related to improvements in areal bone mineral density (aBMD) and strength at loaded sites [8]. The type of sport due to its predominant characteristics can influence skeletal development differently [9], and even suppose a risk factor for low bone mass [10]. In fact, participation in osteogenic sports during childhood, such football, handball or basketball, is associated with a higher aBMD compared with the practice of non-osteogenic sports, like swimming [9]. Furthermore, adolescents engaged in football have shown enhanced aBMD and hip geometry compared with those engaged in swimming and cycling [11]. However, there is a lack of longitudinal data to determine the factors affecting bone development in these groups.

Cardiorespiratory and muscular fitness have been positively associated with bone outcomes (including hip geometry) in active adolescents [4], but the contribution seems to be a function of lean mass. Lean mass plays an important function in the development of aBMD and hip geometry [12], according to the mechanostat theory [13], as the development of the muscles produces a higher tension on the bones. Although the role of lean mass is clear, the association between fat mass and bone mass is debated. Recent studies have shown that the possible association between fat mass and bone mass is completely annulled once the effect of lean mass is controlled [14, 15].

Most studies to date have used dual-energy X-ray absorptiometry (DXA) to evaluate aBMD due to the low radiation and low cost compared to other techniques [16]. There are few studies using hip structural analysis (HSA) to assess bone geometry estimates at the clinically relevant site of the femoral neck (FN) in adolescents [17]. HSA is a technique that uses the properties of DXA images to derive hip geometry estimates that are associated with bone strength [18]. The HSA program measures not only the aBMD of the hip bone but also structural geometry of cross sections traversing the proximal femur at specific locations. To the best of our knowledge, there is only one previous study in adolescent athletes using the recently developed trabecular bone score (TBS) [19]. TBS provides an indirect index of trabecular microarchitecture that is independent of aBMD and was designed to predict fracture risk and fragility of the lumbar spine (LS) [20].

To the best of our knowledge, there are not longitudinal studies investigating the determinants of bone outcomes in adolescent male athletes. Thus, the aim of this study is to identify the determinants of 12-month changes on bone outcomes (aBMD, hip geometry estimates and TBS) in adolescent male athletes.

## Methods

### Study design and participants

The present study shows a 12-month longitudinal analysis of sport participation as part of the longitudinal PRO-BONE (effect of a PROgram of short bouts of exercise on BONE health in adolescents involved in different sports) study, whose purpose, methodology and inclusion/exclusion criteria have been described in detail elsewhere [21]. For the current study, data obtained at baseline (T0) during autumn/winter 2014–15 and follow-up (T1) during autumn/winter 2015–2016 were used (mean difference of visits = 372 days).

After exclusion of three participants who dropped out from the study before T1, the study sample was composed by 104 adolescent male athletes originally recruited from athletic clubs in the South West of England (12–14 years old at baseline): 39 swimmers, 37 footballers (or soccer players) and 28 cyclists. Inclusion criteria were adolescent males 12–14 years old, engaged (≥ 3 h/week) in osteogenic (football) and/or non-osteogenic (swimming and cycling) sports for the last 3 years or more. This criteria was based on previous research demonstrating osteogenic benefits with 3 h of activity per week among adolescents [9]. The exclusion criteria were participation in another clinical trial, any acute infection lasting until < 1 week before inclusion, medical history of diseases or medications affecting bone metabolism or the presence of an injury and non-Caucasian participants.

Written informed consent and assent forms were signed from parents and participants accordingly and all participants completed both visits at the research centre. The methods and procedures of the study have been checked and approved by (1) the Ethics Review Sector of Directorate-General of Research (European Commission, ref. number 618496), (2) the Sport and Health Sciences Ethics Committee (University of Exeter, ref. number 2014/766) and (3) the National Research Ethics Service Committee (NRES Committee South West–Cornwall & Plymouth, ref. number 14/SW/0060).

### Anthropometry and sexual maturation

Body mass (kg) and stature (cm) were measured by using a stadiometer (Harpenden, Holtain Ltd., Crymych, UK) and an electronic scale (Seca 877, Seca Ltd., Birmingham, UK), respectively. Body mass index (BMI, kg/m^2^) from each participant was calculated from these measures and calculated as: body mass (kg)/stature^2^ (m).

Predicted maturity offset, defined as the time before or after peak high velocity was used as a maturational landmark [22]. Maturity offset was calculated for each participant using a validated algorithm in healthy children as follows [23]: − 7.999994 + (0.0036124 × (age × stature in cm)); where *R*^2^ = 0.896 and standard error of the estimate = 0.542.

### Objectively measured physical activity

Physical activity was measured for seven consecutive days using validated accelerometers (GENEActiv, GENEA, UK) [24]. Participants were instructed to place the accelerometer on their non-dominant wrist and data was collected at 100 Hz. In addition, participants logged bedtime, wake up time and every time the device was removed. At least 3 days of recording (including both week and weekend days) with a minimum of 12-h registration per day was set as an inclusion criterion. Data were analysed using 1-s epoch. The time spent in moderate-vigorous physical activity (MVPA) was calculated using a cut-off point of ≥ 1140 counts per minute that has previously been validated in youth [25].

### Dual energy X-ray absorptiometry

A Lunar Prodigy DXA scanner (GE Healthcare Inc., Wisconsin, USA) was used to measure aBMD (g/cm^2^), fat mass (g) and lean mass (g) at specific regions of the body. Four scans were performed to obtain data for the whole body, LS (L1–L4) and dual hip scans. All DXA scans and subsequent in-software analyses were completed by the same researcher, using the same DXA scanner and the enCORE software version 14.10.022 (GE Healthcare Inc., Wisconsin, USA). Despite the coefficient of variation was not determined in the present study, precision studies in paediatric population have shown DXA’s coefficient of variations of 0.74% for total body less head (TBLH) aBMD and 0.64% for LS aBMD in 14–16 years late teens [26].

### Hip structural analysis

The hip geometry estimates at the FN were determined using HSA software and the following variables were obtained: (1) the cross-sectional area (CSA, mm^2^), which is the total bone surface area of the hip excluding the soft tissue area and the trabecular bone; (2) the section modulus (*Z*, mm^3^), which is an indicator of maximum bending strength in a cross section; and (3) the cross-sectional moment of inertia (CSMI, mm^4^), which is an index of structural rigidity and reflects the distribution of mass in the centre of a structural element. The coefficients of variation of these variables have been reported in previous studies and range from 7.9 to 11.7% [27]. A repositioning wedge was used in order to keep the position of the hip joint neutral and obtain an appropriate FN angle. This is key to optimise reproducibility of the hip aBMD and HSA parameters.

### Trabecular bone score

TBS is a DXA-based technological tool that provides an index of bone microarchitectural texture in the LS that predicts fracture risk independently of aBMD [28]. TBS assesses DXA images of the LS scans using a grey-level analysis as the slope at the origin of the log-log representation of the experimental variogram [28]. All TBS analyses were performed by the same trained researcher using the TBS iNsight Software (Medimaps, research version 1.8, Pessac, France). The coefficients of variation of TBS in relation to BMD ranges between 1.1 and 1.4% [20].

### Biochemical analysis: bone and nutritional markers

Capillary blood samples were collected at non-training weekends in the morning in heparin fluoride-coated microvettes (CB 300 tubes, Sarstedt Ltd., Leicester, UK) and centrifuged at 3000 rpm for 15 min at 4 °C. Serum samples were stored at − 80 °C until analysis in a single session. Total serum levels of PINP, CTX-I, 25(OH)D and total calcium were analysed following guidelines [29]. ELISA kits (Abbexa Ltd., Cambridge, UK) for PINP (test range, 6–400 pg mL^−1^; sensitivity, 1.2 pg mL^−1^, inter and intra-assay coefficients of variation, 8.6 and 9.1% respectively); CTX-I (test range, 0.1–7.0 ng mL^−1^, sensitivity, 0.03 ng mL^−1^, inter and intra-assay coefficients of variation, 8.3 and 9.2% respectively) and 25(OH)D (test range, 3–80 ng mL^−1^, sensitivity, 1.2 ng mL^−1^, inter and intra-assay coefficients of variation, 6.4 and 8.0% respectively) were used. Total calcium serum was measured using direct colorimetric assay (Cayman Chemical Company, MI, U.S.A.) and had a sensitivity of 0.25 mg dL^−1^, and the absorbance was read at 570–590 nm (inter and intra-assay coefficients of variation: 7.9 and 9.0% respectively).

### Physical fitness

The fitness tests used in the present investigation have been shown to be reliable and valid in youth [30]. A counter movement vertical jump (cm) was used to provide an estimate of lower limb muscular fitness at least 30 min before performing the 20-m shuttle run test and following a standardised warm up. It was performed using a jump mat (Probotics Inc., Alabama, USA), which calculates the height of the jump based on flight time. Each participant performed three maximal jumps and the best performance was used for the analysis.

Cardiorespiratory fitness was evaluated using the 20-m shuttle run test and was completed in the same sports hall at T0 and T1. The participants were asked to run between two lines set 20 m apart by following the pace of the audio signals produced from a CD player. All participants were equally encouraged to continue the test until they reached a maximal effort. The test ended when the participants failed to reach the line on two consecutive occasions, and the count of the last completed shuttle run was recorded.

### Statistical analyses

Data were analysed using SPSS version 22.0 for Windows (IBM Corp, New York, USA) and descriptive data are reported as mean and standard deviation (SD). The normal distribution of the raw variables and of the regression model residuals was checked and verified using Shapiro-Wilk’s test, skewness and kurtosis values, visual check of histograms, Q-Q and box plots. Collinearity was checked for the variables using the variance inflation factor (VIF). Descriptive analysis was obtained by (1) one way analysis of variance (ANOVA) with Bonferroni post hoc comparisons to detect differences between groups and (2) ANOVA with repeated measures to analyse the differences between T0 and T1 within each group.

Type of sport, changes (Δ, T1-T0) in maturity offset, stature, BMI, lean mass, fat mass, vertical jump, cardiorespiratory fitness, MVPA, 25(OH)D, calcium, CTX and PINP were selected as predictors based on their relationship with bone outcomes [5, 12, 31, 32]. Multiple linear regression analyses were used to examine the contribution of the change in each predictor (Δ) on the change in bone outcomes (Δ). Baseline bone outcomes were controlled in all linear regressions following previous studies [33]. In addition, a dummy variable for type of sport was computed (footballers, swimmers and cyclists) with footballers as the reference group. The standardised regression coefficients (*β*) significance was set at alpha level of 5%. The squared semi-partial correlation coefficients (sr^2^) were used to determine the contribution of each predictor in the overall variance of the model after removing shared contributions with other predictors. In addition, the effect size (Cohen’s *f*^2^, ES) was calculated following the method proposed by Cohen [34].

## Results

Table 1 shows the raw descriptive characteristics of the participants at T0 and T1. Briefly, within-group differences show that most variables significantly changed over time except fat mass, weekly training hours, serum calcium levels and 25(OH)D. Taking footballers as the reference group, between-group differences showed that swimmers were significantly older, more mature, taller, heavier and had more lean mass; and less 25(OH)D, cardiorespiratory fitness and MVPA at T0 and T1. In addition, cyclists trained less hours per week and had poorer cardiorespiratory fitness at T0 and T1.

**Table 1:** Descriptive characteristics of the participants at baseline and after 1 year of sport participation

	Swimmers (*N* = 39)	Footballers (*N* = 37)	Cyclists (*N* = 28)	All groups (*N* = 104)
Age (years)				
T0	13.5 (1.0)	12.9 (0.9)^a^	13.3 (1.1)	13.2 (1.0)
T1	*14.6 (1.0)*	*13.9 (1.0)* ^a^	*14.2 (1.0)*	*14.3 (1.0)*
Maturity offset (years)				
T0	0.1 (1.0)	− 0.8 (0.8)^a^	− 0.3 (1.1)	− 0.3 (1.0)
T1	*1.1 (0.9)*	*0.2 (1.0)* ^a^	*0.6 (1.1)*	*0.6 (1.1)*
Stature (cm)				
T0	164.9 (9.6)	155.2 (9.3)^a^	161.2 (10.7)	160.4 (10.6)
T1	*171.4 (8.7)*	*162.7 (10.3)* ^a^	*167.4 (10.4)*	*167.2 (10.4)*
Body mass (kg)				
T0	51.8 (8.5)	44.3 (7.9)^a^	48.8 (11.8)	48.3 (9.7)
T1	*58.5 (8.1)*	*50.8 (9.7)* ^a^	*54.7 (12.5)*	*54.7 (10.4)*
BMI (kg/m^2^)				
T0	18.9 (1.6)	18.3 (1.4)	18.6 (3.0)	18.6 (2.0)
T1	*19.8 (1.7)*	*19.0 (1.8)*	*19.3 (3.0)*	*19.4 (2.2)*
Total lean mass (kg)				
T0	41.0 (8.9)	35.4 (7.2)^a^	37.5 (7.5)	3.8 (8.2)
T1	*47.7 (8.5)*	*41.2 (9.2)* ^a^	*42.9 (8.2)*	*4.4 (9.1)*
Total fat mass (kg)				
T0	8.2 (3.3)	6.6 (2.4)	8.7 (7.3)	7.8 (4.5)
T1	7.8 (3.2)	6.9 (2.7)	8.9 (7.9)	7.8 (4.8)
Training (h/week)				
T0	9.5 (5.0)^b^	10.0 (2.3)^b^	5.2 (2.1)	8.5 (4.1)
T1	9.0 (3.5)^b^	9.4 (1.7)^b^	5.6 (2.0)	8.2 (3.0)
MVPA (min/day)				
T0	*85.0 (30.9)*	*119.8 (29.7)* ^a^	*106.5 (33.7)* ^a^	*103.5 (34.4)*
T1	62.9 (21.8)	92.4 (25.7)^a^	85.6 (21.8)^a^	79.0 (27.1)
Vertical jump (cm)				
T0	42.3 (7.1)	41.4 (6.0)	40.9 (6.9)	41.6 (6.6)
T1	*46.7 (8.1)*	*43.5 (6.3)*	*43.6 (6.7)*	*44.7 (7.2)*
Cardiorespiratory fitness (shuttle)				
T0	68.7 (20.1)	82.9 (17.6)^a,b^	69.1 (21.4)	73.9 (20.5)
T1	*78.1 (20.6)*	*90.7 (19.4)* ^a,b^	*83.8 (21.3)*	*84.1 (20.9)*
Calcium (mg/dl)				
T0	10.0 (0.5)	10.0 (0.4)	10.0 (0.4)	10.0 (0.4)
T1	9.9 (0.5)	10.0 (0.4)	9.9 (0.3)	9.9 (0.4)
25(OH)D (ng/ml)				
T0	13.7 (1.2)	14.4 (1.6)^a^	14.4 (0.6)	14.2 (1.3)
T1	13.4 (0.9)	15.2 (1.2)^a^	14.9 (1.1)^a^	14.4 (1.3)
PINP (pg/ml)				
T0	*355.5 (9.9)*	352.0 (13.5)	*350.8 (2.8)*	*353.0 (10.3)*
T1	335.8 (17.6)	346.6 (18.2)^a,b^	327.2 (14.2)	337.3 (18.5)
CTX (ng/ml)				
T0	1.7 (0.3)	1.6 (0.3)^b^	1.8 (0.2)	1.7 (0.3)
T1	*1.8 (0.2)*	*1.9 (0.1)*	*1.9 (0.1)*	*1.9 (0.1)*

Values presented as mean (SD)

*BMI* body mass index, *LM* lean mass, *MVPA* moderate to vigorous physical activity, *25(OH)D* 25-hydroxyvitamin D, *T0* baseline values, *T1* 1-year values

Superscript letters denote a significant difference (*p* < 0.05) compared to: ^a^(swimmers), ^b^(cyclists)

Significant differences between T0 and T1 of each sport are in italics (*p* < 0.05)

In Table 2, multivariate regression models significantly explained 38–60% of the variance in the change of aBMD outcomes (ES = 0.61–1.50) over 12 months. Δregion-specific lean mass was the most consistent predictor of changes in aBMD outcomes (*β* = 0.591 to 0.696), followed by cycling participation (*β* = − 0.233 to − 0.262), swimming participation (*β* = − 0.315 to − 0.336) and ΔMVPA (*β* = 0.165).

**Table 2:** Multiple regression models for aBMD in adolescent male athletes

ΔPredictors		*β*	sr^2^	*P*		ΔPredictors	*β*	sr^2^	*P*
		STD	values	values			STD	values	values
ΔTBLH aBMD (**R**^**2**^ = **0.60)**	Swimmers	− 0.239	0.017	0.070	ΔHip aBMD (**R**^**2**^ = **0.55)**	Swimmers	− 0.315	0.028	**0.027**
	Cyclists	− 0.233	0.027	**0.022**		Cyclists	− 0.262	0.031	**0.019**
	Maturity offset	0.242	0.006	0.256		Maturity offset	0.068	0.001	0.750
	Stature	0.029	0.000	0.903		Stature	0.048	0.000	0.828
	BMI	− 0.086	0.002	0.567		BMI	0.019	0.000	0.907
	Total lean mass	0.591	0.031	**0.015**		Legs lean mass	0.632	0.037	**0.012**
	Total fat mass	0.249	0.012	0.116		Legs fat mass	0.145	0.004	0.391
	Vertical jump	0.004	0.000	0.964		Vertical jump	0.039	0.001	0.637
	Cardiorespiratory fitness	− 0.005	0.000	0.953		Cardiorespiratory fitness	0.070	0.004	0.412
	MVPA	0.165	0.024	**0.030**		MVPA	0.151	0.020	0.059
	Calcium	− 0.028	0.001	0.741		Calcium	0.050	0.002	0.578
	25(OH)D	0.086	0.006	0.268		25(OH)D	0.042	0.001	0.605
	CTX	− 0.035	0.001	0.670		CTX	− 0.024	0.000	0.781
	PINP	− 0.078	0.004	0.366		PINP	− 0.062	0.003	0.495
ΔLS aBMD (**R**^**2**^ = **0.58**)	Swimmers	0.051	0.001	0.676	ΔFN aBMD **(R**^**2**^ **= 0.38)**	Swimmers	− 0.336	0.033	**0.042**
	Cyclists	− 0.037	0.001	0.706		Cyclists	− 0.261	0.033	**0.040**
	Maturity offset	− 0.197	0.005	0.350		Maturity offset	0.232	0.007	0.360
	Stature	0.355	0.013	0.111		Stature	− 0.484	0.026	0.068
	BMI	0.051	0.001	0.737		BMI	0.044	0.000	0.815
	Trunk lean mass	0.597	0.034	**0.012**		Legs lean mass	0.696	0.044	**0.020**
	Trunk fat mass	0.114	0.003	0.480		Legs fat mass	0.080	0.001	0.688
	Vertical jump	0.070	0.004	0.387		Vertical jump	0.028	0.001	0.777
	Cardiorespiratory fitness	− 0.081	0.005	0.331		Cardiorespiratory fitness	0.171	0.022	0.093
	MVPA	0.127	0.014	0.099		MVPA	0.138	0.017	0.144
	Calcium	− 0.021	0.000	0.809		Calcium	0.050	0.002	0.637
	25(OH)D	0.113	0.011	0.155		25(OH)D	0.065	0.003	0.506
	CTX	− 0.099	0.007	0.253		CTX	0.035	0.001	0.733
	PINP	− 0.151	0.015	0.088		PINP	− 0.007	0.000	0.947

Bold numbers denote a significant difference (*p* ≤ 0.05)

*β* standardised regression coefficient, *sr*^2^: squared semi-partial correlation coefficients, *aBMD* areal bone mineral density, *TBLH* total boy less head, *LS* lumbar spine, *FN* femoral neck, *BMI* body mass index, *LM* lean mass, *MVPA* moderate-to-vigorous physical activity, *25(OH)D* 25-hydroxyvitamin D

In Table 3, multivariate regression models significantly explained 36–62% of the variance in the change of hip geometry estimates (ES = 0.56–1.63) and 45% of the variance in the change of TBS (ES = 0.82) over 12 months. Cycling participation was the most consistent predictor of changes in hip geometry estimates (*β* = − 0.174 to − 0.268), followed by Δregion-specific lean mass (*β* = 0.587) and Δcardiorespiratory fitness (*β* = 0.253). Finally, cycling and swimming participation (*β* = − 0.347 to − 0.453), Δregion-specific lean mass (*β* = 0.848) and Δstature (*β* = 0.720) were predictors of change in TBS. ΔMaturity offset, ΔBMI, ΔFat mass, Δvertical jump, Δcalcium, Δ25(OH)D, ΔCTX and ΔPINP were not associated with changes in bone outcomes at any skeletal site after accounting for the other predictors.

**Table 3:** Multiple regression models for HSA and TBS in adolescent male athletes

ΔPredictors		*β*	sr^2^	*P*	ΔPredictors		*β*	sr^2^	*P*
		STD	values	values			STD	values	values
ΔCSA	Swimmers	− 0.025	0.000	0.874	Δ**Z**	Swimmers	− 0.171	0.010	0.248
**(R**^**2**^ **= 0.36)**	Cyclists	− 0.205	0.022	0.100	**(R**^**2**^ **= 0.41)**	Cyclists	− 0.268	0.038	**0.024**
	Maturity offset	− 0.370	0.016	0.162		Maturity offset	0.086	0.001	0.734
	Stature	0.450	0.018	0.130		Stature	− 0.067	0.000	0.816
	BMI	0.016	0.000	0.935		BMI	− 0.194	0.008	0.305
	Legs lean mass	0.364	0.011	0.245		Legs lean mass	0.587	0.027	**0.050**
	Legs fat mass	0.182	0.006	0.371		Legs fat mass	0.313	0.019	0.112
	Vertical jump	− 0.127	0.013	0.201		Vertical jump	− 0.024	0.000	0.804
	Cardiorespiratory fitness	0.253	0.049	**0.015**		Cardiorespiratory fitness	0.157	0.019	0.112
	MVPA	0.137	0.016	0.154		MVPA	0.098	0.008	0.292
	Calcium	− 0.127	0.011	0.234		Calcium	0.096	0.006	0.348
	25(OH)D	0.064	0.003	0.509		25(OH)D	− 0.007	0.000	0.940
	CTX	0.099	0.007	0.333		CTX	0.018	0.000	0.858
	PINP	0.051	0.002	0.638		PINP	− 0.048	0.002	0.645
ΔCSMI	Swimmers	− 0.148	0.008	0.205	ΔTBS	Swimmers	− 0.453	0.068	**0.002**
**(R**^**2**^ = **0.62)**	Cyclists	− 0.174	0.016	**0.047**	**(R**^**2**^ **= 0.45)**	Cyclists	− 0.347	0.056	**0.005**
	Maturity offset	0.158	0.003	0.430		Maturity offset	0.239	0.008	0.297
	Stature	0.222	0.005	0.313		Stature	0.720	0.069	**0.002**
	BMI	− 0.040	0.000	0.791		BMI	− 0.216	0.010	0.224
	Legs lean mass	0.279	0.007	0.241		Trunk lean mass	0.848	0.069	**0.002**
	Legs fat mass	0.176	0.006	0.261		Trunk fat mass	0.228	0.010	0.222
	Vertical jump	− 0.053	0.002	0.493		Vertical jump	− 0.049	0.002	0.590
	Cardiorespiratory fitness	0.093	0.007	0.237		Cardiorespiratory fitness	− 0.042	0.001	0.663
	MVPA	0.128	0.014	0.088		MVPA	− 0.107	0.010	0.229
	Calcium	0.032	0.001	0.699		Calcium	− 0.041	0.001	0.680
	25(OH)D	0.014	0.000	0.857		25(OH)D	0.001	0.000	0.991
	CTX	− 0.006	0.000	0.943		CTX	0.015	0.000	0.879
	PINP	0.011	0.000	0.900		PINP	− 0.126	0.011	0.216

Bold numbers denote a significant difference (*p* ≤ 0.05)

*β* standardised regression coefficient, *sr2* squared semi-partial correlation coefficients, *CSA* cross-sectional area, *CSMI* cross-sectional moment of inertia, *Z* section modulus, *TBS* trabecular bone score, *BMI* body mass index, *LM* lean mass, *CRF* cardiorespiratory fitness, *MVPA* moderate-to-vigorous physical activity, *25(OH)D* 25-hydroxyvitamin D

## Discussion

To the best of our knowledge, this is the first longitudinal study that investigates the determinants of change in aBMD, hip geometry estimates and TBS in adolescent male athletes. The main findings from the present study are (1) region-specific lean mass was the most explanatory variable of changes in aBMD outcomes and (2) the practice of low impact sports came out as a strong and negative predictor of change in aBMD (both cycling and swimming), hip geometry estimates (cycling) and TBS (both).

The variance explained by the determinants ranged from 38 to 60% for aBMD outcomes, 36–62% for hip geometry estimates and 45% for TBS. In our previous cross-sectional study, we reported that the significant determinants explained 49–75% of the variance in aBMD outcomes and 72–78% of the variance in hip geometry estimates [12]. The longitudinal associations described in this study reflect relationships within individuals over 12 months, which represents an advantage over cross-sectional studies in which accounting for duration of exposure to predictors is not feasible.

In this study, the strongest predictor of changes in aBMD (TBLH, LS, hip and FN) and one of the predictors of changes in hip geometry estimates (*Z*) and TBS was Δregion-specific lean mass. This agrees with our previous cross-sectional investigation, in which region-specific lean mass was the strongest determinant of aBMD at TBLH, LS, legs and arms and hip geometry estimates (CSMI and *Z*) [12]. Similarly, a previous cross-sectional study indicated that total lean mass may be an important determinant of total body aBMD and LS aBMD in non-athletic children [35]. Evidence from longitudinal studies has shown that changes in lean mass are strongly associated with changes in aBMD at LS and hip, CSA and whole body bone mineral content in pre-adolescent inactive females [36–38]. These results are similar to our findings despite the fact that we used region-specific lean mass due to the demonstrated specific adaptations of the skeleton site in response to external loading [12]. Our results agree with a previous study in school children in which lean mass was found as a predictor of TBS [39].

In the present study, the type of sport came up as another strong (but negative) predictor of change in aBMD outcomes (TBLH, hip and FN), hip geometry estimates (CSMI and *Z*) and TBS. More specifically, the practice of cycling predicted most of the changes observed in aBMD and hip geometry estimates. In addition, swimming participation was negatively associated with aBMD outcomes (hip and FN) and TBS. These two non-weight bearing sports are considered non-osteogenic due to the lack of impact resulting from ground reaction forces [40, 41] and, therefore, they do not positively affect bone mass during adolescence. According to our cross-sectional comparisons between sport groups, swimmer and cyclist male adolescents had less aBMD and hip geometry estimates compared to those involved in an osteogenic sport like football, and similar bone outcomes compared to an active control group [11]. In the same line, elite female adolescent swimmers presented lower aBMD compared to footballers, supporting our findings [17].

Our results showed that Δstature was positively associated with changes only in TBS. Previous cross-sectional evidence showed that stature was positively associated with aBMD outcomes in young athletic and non-athletic population [12, 35]. Differences among studies can be due to the fact that TBS is independent of aBMD [20] and also to the different study designs. To the best of our knowledge, this is the first follow-up study reporting determinants of TBS change and, therefore, our results are not comparable with previous research. In addition, we found MVPA as another positive predictor of changes in TBLH aBMD, which is in line with previous evidence in growing population [42, 43]. Finally, Δcardiorespiratory fitness was positively associated with changes in CSA in this study. A recent cross-sectional study, albeit in young overweight and obese men, showed that VO_2_ max (directly measured) was significantly correlated with hip geometry outcomes such as CSA and *Z* [44]. The hip is a sensible site to external loading [27] and the lower limbs are key to perform the proposed cardiorespiratory fitness test. This finding may have clinical implications in reducing the risk of future hip osteopenia and/or osteoporosis.

The combination of DXA, HSA software, TBS analysis and biochemical markers provides a more comprehensive insight of the changes in bone outcomes. To date, the number of studies using TBS in paediatric population is very limited and the findings from this study will help identifying predictors of TBS change in young athletic population. In addition, all participants presented mild-to-moderate 25(OH)D deficiency (at T0 and T1) as defined by a threshold between 10 and 19 ng/ml [45]. This study allows us to investigate the determinants of change in bone outcomes, including the type of sport. In this regard, the inclusion of an inactive control group to compare with would have been of scientific interest. In addition, the number of participants is relatively small and this should be taken into account when interpreting the results. Finally, the 20-m shuttle run test has been used to assess cardiorespiratory fitness, which may underestimate cyclists and swimmers’ aerobic capacity, as they were not necessarily familiar with this type of activity. However, this test has been used worldwide in children and adolescents and it has been shown to be reliable and valid [30].

In conclusion, this study provides evidence that changes in region-specific lean mass and the type of sport are the most consistent determinants of 12-month changes in aBMD, hip geometry estimates and TBS in adolescent male athletes. Despite the practice of swimming and cycling seems not to be beneficial for bone changes, its combination with high impact and weight-bearing activities such as plyometric jumps (REF) is recommended. These findings may help researchers in identifying and considering key predictors of bone change in their longitudinal studies with young athletic population.

## Abbreviations

aBMD: areal bone mineral density
BMI: body mass index
CSA: cross-sectional area
CSMI: cross-sectional moment of inertia
DXA: dual energy X-ray absorptiometry
FN: femoral neck
HSA: hip structural analysis
LS: lumbar spine
MVPA: moderate to vigorous physical activity
TBLH: total body less head
TBS: trabecular bone score
Z: section modulus
25(OH)D: 25-hydroxyvitamin D
